# *Schistosoma mansoni* infection causes consistent changes to the fecal bacterial microbiota of mice across and within sites

**DOI:** 10.1371/journal.pone.0324638

**Published:** 2025-05-30

**Authors:** Mariam A. Mhanna, David T. Gauthier, Lisa M. Shollenberger

**Affiliations:** 1 Department of Biological Sciences, College of Sciences, Old Dominion University, Virginia, United States of America; 2 Department of Infectious Diseases, College of Veterinary Medicine, University of Georgia, Georgia, United States of America; Beni Suef University Faculty of Veterinary Medicine, EGYPT

## Abstract

Eggs of *Schistosoma mansoni* are produced by adult female worms in mesenteries of infected hosts. Eggs can cross the intestinal barrier and form granulomas in the tissue or breach and exit the host through fecal excretion. These interactions may affect the host microbiome assemblages. Given the potential for schistosomal alteration of host gut microbiome and subsequent effects on the fecal bacterial composition, it is important to conduct controlled microbiome studies on model animals. While pursuing these studies, it is important to take into account the different conditions in which microbiome studies are conducted and their consequent impacts on variability and reproducibility of results. In particular, we are interested in inter-institutional effects on controlled microbiome studies, in which the study location itself may impact study outcomes. In this work, we report global changes caused by acute and chronic schistosomiasis on the fecal microbiome of mice at two different institutions and three timepoints.

## Introduction

The fecal microbiome is composed of a diverse collection of fungi, protozoa, bacteria, archaea, and viruses that act as a representation of the population residing in the gut of the respective hosts [1,2]. The human microbiome contains an estimated 10–100 trillion microbial cells, with the bulk of diversity represented by intestinal microbiota [2]. The fecal microbiome plays a pivotal role in preserving the integrity of the gut barrier, metabolism of nutrients, interactions with the hosts’ immune systems, and the facilitation of the gut-brain axis.

Schistosomiasis is a neglected tropical disease that affects approximately 250 million people worldwide primarily in developing areas such as Sub-Saharan Africa and Southeast Asia [3]. *Schistosoma mansoni* infects humans when present in a body of freshwater [4]. The eggs excreted in feces from the host hatch and release miracidia that penetrate the tegument of the intermediate host, snails (*Biomphalaria* spp). In snails, miracidia metamorphose into sporocysts that asexually reproduce into cercariae which are the infectious agent of schistosomiasis [4,5]. Cercariae are released from the snail into the water and penetrate the mammalian host, at which point the cercariae will shed their tail to become circulating schistosomula. Schistosomula migrate to the liver by portal blood then mature into adult female and male worms. The worms pair and migrate to mesenteric venules of the bowel/rectum and sexually produce eggs [5]. Eggs breach the serosal intestinal wall and form granulomas that aid in the extrusion in the intestinal lumen [6].

The fecal microbiome can be affected by several abiotic and biotic factors. One of which being infections in the host, including helminths infections [7]. *Schistosoma* worms, through their production of eggs in the host as well as other systemic changes, have been shown to cause imbalances in the fecal microbiome composition. The link between *Schistosoma spp.* and the fecal microbiome occurs when the eggs breach the host’s intestinal wall as described above. This action contributes to affecting the fecal microbiome of the host and whether this effect is variable or conserved in different studying sites is unclear.

According to the Human Microbiome Project, the composition of microbiomes is affected by several factors [8]. These include diet, genetic factors, geographic location, and antibiotic usage [8]. As for microbiome studies, differences like cages, food, and/or animal facilities may affect the reproducibility and rigor in performing such studies. The culmination of biotic and abiotic factors and the overall effect they have on causing imbalances in the fecal microbiome leads to a lot of variabilities and challenges in performing microbiome-related studies.

There still is discord in the literature regarding the variation of findings relating to this data. The variability of microbiome studies and the ability to replicate and still observe comparable results is often challenging and requires a lot of rigor and consistency. In this paper, we discuss the fecal microbiome’s composition and diversities compared between healthy BALB/c mice and those infected with *S. mansoni*, at acute and chronic stages, as performed at two independent institutions at different times.

## Materials and methods

### Ethics statement

All manipulations were performed in accordance with the Institutional Biosafety Committee (UGA 2014-0016, ODU 18-030, ODU 19-005) and the Institutional Animal Care and Use Committee (UGA A2015-04017, ODU 19-021). Studies have been performed in accordance with applicable ethical standards and regulations. All ODU laboratory personnel working with live mice are required to complete the following CITI trainings every 3 years: (1) Working with the IACUC, (2) Reducing Pain and Distress in Laboratory Mice and Rats, and (3) Working with Mice in Research.

### Mice groupings

Six-week-old female BALB/c mice were purchased from Envigo (Indianapolis, IN) and housed at the animal facilities of University of Georgia (UGA) or Old Dominion University (ODU). Three separate experiments were performed: the first at UGA in 2017, followed by ODU1 in 2019 and ODU2 in 2020. Mice in the three experiments (UGA, ODU1, ODU2) were housed in the animal facilities under specific pathogen-free conditions and allowed to acclimate for 1 week prior to any experimental manipulations at the time of the experiments. Uninfected mice groups were housed separately from infected mice. Each cage has an n = 3 mice at UGA and n = 4 or 5 at ODU1 and ODU2. Table 1 shows the total number of mice in each experiment as well as the number of mice that had their fecal samples sequenced. Mice were identified by ear punch or ear tag (RapID, San Francisco).

**Table 1:** Group distribution of uninfected and infected mice groups. Total number of mice in each of the three experiments (UGA, ODU1, and ODU2) and their cage distributions.

	UGA	ODU1	ODU2
total mice per experiment	18	27	30
total uninfected mice	9	12	15
total *S. mansoni* infected mice	9	15	15
Total number of cages	6	6	6
uninfected mice housed per cage	3	4	5
infected mice housed per cage	3	5	5
mice sequenced per cage	1	3	3
total mice sequenced	6	18	18
total uninfected mice sequenced	3	9	9
total infected mice sequenced	3	9	9

### Schistosome infection of mice

Infectious cercariae were obtained by direct light exposure of PR-1 strain *S. mansoni*-infected *Biomphalaria glabrata* snails (received from BRI). Cercariae (30–35) were injected intraperitoneally in half of the mice to induce a low-level schistosome infection while the other half was left uninfected.

These experiments were approved by IACUC as Category C, meaning there is no more than momentary or slight pain or distress and no use of pain-relieving drugs, or no pain or distress, as we don’t expect to see pain or distress in the animals infected with *S. mansoni*. Animal resource personnel observed the animals daily. If animals displayed certain severe clinical symptoms such as lethargy, anorexia (appear underweight), visible spine or pelvis, the mice would either be immediately euthanized or would immediately begin a monitoring program. If not euthanized, lab personnel would monitor the mice daily. The criteria for humane euthanasia included: (1) 20% weight loss compared to naive age-matched mice (based on published, strain-specific body weight charts), (2) lethargy that lasts 48 hours, or (3) a combination of 15% weight loss and lethargy that lasts 36 hours. As expected, no mice entered the monitoring program, and no mice died during the 10-week low-level infection of this study.

### Samples collection

Blood and fecal samples were individually collected from mice at both institutions. Blood was collected 8 weeks post-infection (wpi) by submandibular bleeding into a Sera-Gel tube (Sarstedt, Newton), centrifuged at 10,000 × *g* at 20°C for 5 minutes to separate sera, then stored frozen until use. Fresh fecal samples were collected longitudinally from each mouse prior to infection, 4 wpi (weeks post infection), 6 wpi, 8 wpi, and 10 wpi and stored at -80°C until use. After fecal collection at 10 wpi, mice were designated into another study.

### SEA/SWAP preparation

*Schistosoma mansoni* PR-1 male worms and eggs were purchased from BRI to prepare soluble male worm antigen proteins (SWAP) and soluble egg antigen (SEA). Worms and eggs were homogenized separately by acid-washed dounce homogenizer. The homogenized liquids were collected and spun at 16000 × *g* for 1 hour. The supernatant was filtered at 0.45 µm into new sterile microfuge tube and stored at -80°C until use.

### Infection confirmation

Mice sera were used for an indirect ELISA. Nunc high-binding ELISA plates (Roskilde, Denmark) were coated with 10µg/ml of the prepared SEA or SWAP in coating buffer (Sodium carbonate at pH 9.6, NaHCO_3_, and Na_2_CO_3_ in 50 ml water). Sera were incubated in the coated plates to confirm the presence of the anti-SWAP and anti-SEA antibodies. Anti-mouse (IgG + IgM)-Horseradish peroxidase (HRP) was used for detection of bound antibodies from the sera, and then detected with TMB substrate for up to 30 minutes for development. The reaction was stopped with sulfuric acid (2N) and the absorbance at 450–570 nm was measured by Biotek nanodrop (Agilent, Santa Clara, CA)

### DNA extraction

Single frozen fecal pellets were weighed using an analytical balance, then DNA was isolated using the DNeasy PowerSoil kit immediately after weighing (Qiagen, Valencia, CA). DNA was quantified at 260 nm using the Epoch 2 machine Take3 plate reader (Agilent, Santa Clara, CA), and diluted to 5 ng/μL with nuclease free water in preparation for 16S rRNA amplification. Samples from UGA were processed separately while samples from ODU1 and ODU2 were processed at the same time.

### 16S rRNA gene sequencing

The 16S rRNA V3-V4 region was amplified using the Illumina rRNA metagenomic sequencing Library Preparation protocol and the Illumina primers (part number 15044223 Rev. B) [9]. PCR was performed to amplify 16S rRNA V3-V4 region using the universal primer set at 1µM [10], with 5 ng/µL of DNA and 2x KAPA HiFi Hotstart Readymix (Roche, South San Francisco CA). PCR products were purified using AmPure XP beads (Beckman Coulter, Brea, CA) and the index PCR was done to add the Nextera XT set A Index primers (Illumina, San Diego, CA). Post PCR cleanup, samples were fluorescently quantified using AccuBlue (Biotium, Fremont, CA) and pooled in equimolar amounts according to the Illumina protocol (part number 15044223 Rev. B). Library size was checked using the Bioanalyzer High Sensitivity DNA Kit (Agilent, Santa Clara, CA). Library was diluted to 10 nM and loaded onto the Illumina MiSeq machine at 10 pM with a 15% PhiX concentration (Illumina, San Diego, CA).

### Data processing

Sequence files (.fastq) were analyzed using Quantitative Insights Into Microbial Ecology (QIIME2) software [11]. Trimming, read filtration, and generation of Amplicon Sequence Variants (ASVs) was performed using DADA2 [12] implemented within QIIME2. Forward and reverse paired-end reads were left-truncated at 240 bp based on evaluation of per-base quality charts. Chimaera filtration was performed at 2-fold overabundance (--p-min-fold-parent-over-abundance 2). The reads were trimmed for the amplicon PCR primers using default settings before merging. Bacterial sequences were annotated using the SILVA 138.1 database [13,14]. Based on alpha-rarefaction curves of Shannon index, samples with fewer than 7500 reads were excluded (2 samples were lost post- filtration) (S1 Fig). Taxonomy-based filtration was performed to remove reads identified as chloroplast and mitochondria Amplicon Sequence Variants. All filtering processes were conducted prior to any downstream analyses. The raw sequence data is available in the Mendeley database https://data.mendeley.com/datasets/dhb6bw6h3j/2.

### Statistical analysis

Alpha diversity was determined as Shannon entropy with the diversity core-metrics-phylogenetic module in QIIME2. Alpha-diversity was analyzed across experiment, infection status, and week post-infection via rank-transformed ANOVA. Assumptions of normality (Shapiro-Wilk), autocorrelation (Durbin-Watson), and homogeneity of variance (Levene) were tested at <alpha > = 0.05. The null hypothesis of deviation from assumptions was not rejected in any case. Beta diversity was calculated as Bray-Curtis dissimilarity and Principal Coordinate analysis (PCoA) was performed in QIIME2. Beta diversity was rarefied to the same level as the rest of the analyses (7500 reads minimum). Outputs from QIIME2 PCoA were imported into RStudio v2024.04.2 + 764 using the read_qza function of package “qiime2R” [15] and plotted with ggplot2. One-way analyses to examine effects of dispersion [16] were performed with the beta-group-comparison tool of QIIME2. Principal Components Analysis (PCA) was performed with the “PLNmodels” package in R [17] using Geometric Mean of Pairwise Ratio (GMPR) offsets [18] and model selection under Bayesian Information Criterion (BIC), and visualized with ggplot2. Raw data files were exported from QIIME2 post samples processing and statistics were performed by GraphPad Prism v9.5.1 or R v4.3.2. GraphPad Prism was used to generate bar plot figures and perform the statistical analyses associated with them. R was used to generate PCAs and perform statistical analyses on alpha diversity box plots, and beta diversity. Findings were considered statistically significant at p < 0.05. *, **, and **** denote p < 0.05, p < 0.01, and p < 0.0001, respectively by one-way ANOVA using Sidak Test.

## Results

### Library statistics

Two samples were excluded with less than 7500 reads based on results from alpha-rarefaction (S1 Fig). The remaining 123 individuals had a minimum and maximum of 9130 and 253034 reads, respectively passing filtration through the DADA2 pipeline. The median read count for these individuals was 43266.

### Effect of *S. mansoni* on the fecal microbiome composition at the phylum level

**Fig 1:**
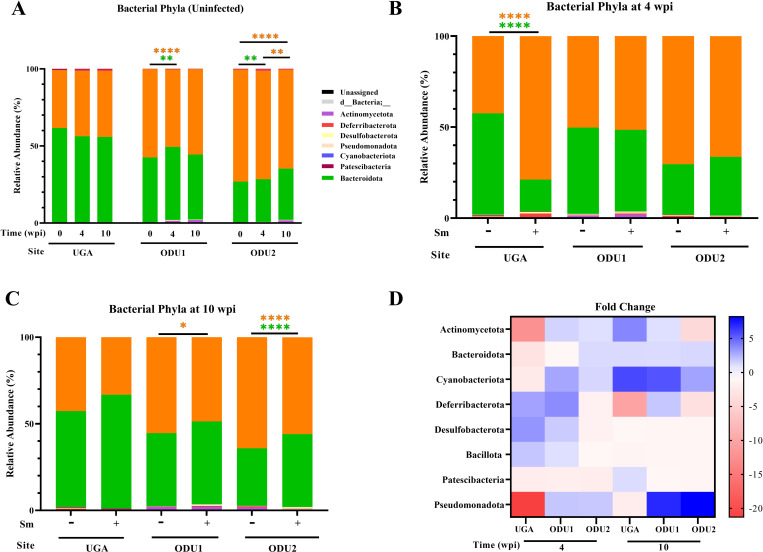
Relative abundances of bacterial phyla detected in fecal samples by bar plots. (A) Bar plots generated using GraphPad Prism, of relative abundance of the major bacterial phyla found in feces of uninfected mice at three different timepoints in three different experiments. Bar plots of relative abundance comparing infected and age-matched uninfected mice in three experiments for acute (B) and chronic (C) timepoints. The heat map of the fold change of the major bacterial phyla in infected mice normalized against the mean of age-matched uninfected groups (D). The value represented by the color gradient is on the right of the figure. Findings were considered statistically significant at p < 0.05. *, **, and **** denote p < 0.05, p < 0.01, and p < 0.0001, respectively by one-way ANOVA using Sidak Test. The color of the significant asterisk indicates the respective phyla.

Eight phyla were identified in all experiments. There were two predominant phyla (Bacteroidota and Bacillota) at both institutions at all times for uninfected and infected mice (Fig 1).

Uninfected mice at UGA had no significant age-related changes of their fecal microbiome composition at the phylum level at all timepoints (Fig 1A). At 4 weeks, ODU1 and ODU2 uninfected mice showed some age-related significant changes, where Bacteroidota increased significantly (ODU1 and ODU2) while Bacillota decreased significantly (ODU1). At 10 weeks, ODU1 had no significant age-related changes in uninfected mice at the phyla level. Whereas, in ODU2, there was a highly significant decrease of Bacillota in the uninfected mice at this stage (Fig 1A).

Infected mice in the acute stage of infection (4 wpi) (Fig 1B) at UGA showed major significant differences in their compositions of Bacillota (+36.1%) and Bacteroidota (-38.22%), compared to age-matched uninfected mice. The profiles at ODU1 and ODU2 are similar between the two institutions, while different from UGA; Bacteroidota and Bacillota had modest increase and decrease respectively (<5%) in infected mice when compared to the uninfected mice, however these trends showed no statistical significance.

Infected mice in the chronic stage of infection (10 wpi) (Fig 1C) in all three experiments, showed a higher presence of Bacteroidota and a lower Bacillota abundance when compared to age-matched uninfected mice, with ODU1 (Bacillota p < 0.05) and ODU2 (Bacteroidota p < 0.0001, Bacillota p < 0.0001).

The comparison of infected to age-matched uninfected mice are presented for acute (4 wpi) and chronic (10 wpi) time points as fold changes (Fig 1D). During acute infections (4 wpi), UGA and ODU1 had some consistent phyla increases in Deferribacterota, Desulfobacterota, and Bacillota when compared to age matched mice. Intrainstitutionally, there were some similar relative abundance increases in Actinomycetota, Cyanobacteriota, and Pseudomonadota at ODU1 and ODU2 when compared to uninfected mice. Finally, Patescibacteria decreased in all three experiments. During chronic infection (10 wpi), there were some consistent changes in phyla compared to age matched uninfected mice between both institutions; Cyanobacteriota increased while Desulfobacterota decreased. The transition from acute to chronic, Cyanobacteriota, Patescibacteria, and Pseudomonadota increased in all three experiments, while Deferribacterota, Desulfobacterota, and Bacillota decreased.

**Fig 2:**
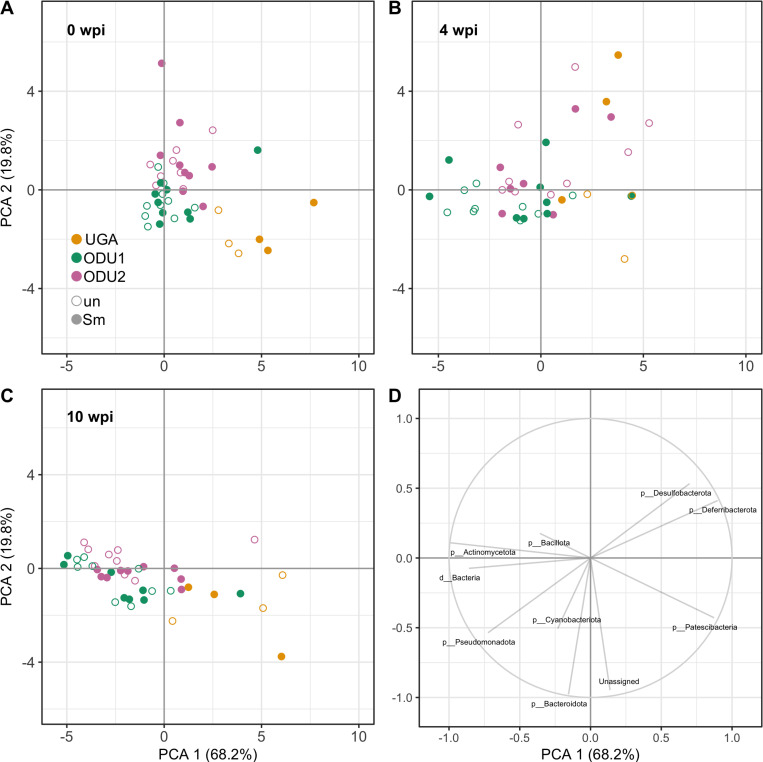
Relative abundances of bacterial phyla detected in fecal samples by Principal Component Analysis. Principal Component Analysis (PCA) based on the relative abundance of bacterial phyla found in the fecal microbiome. Infected (Sm- filled circle) and uninfected (un- hollow circle) mice at 0 wpi (A), 4 wpi (B), and 10 wpi (C) are shown. UGA (orange), ODU1 (blueish green), and ODU2 (reddish purple). (D) Vectors for major phyla (all weeks).

Principal Components Analysis at the phylum level are presented in Fig 2. Effects of site are most notable in the clustering of individual data points, with mice from UGA clustering in the upper right quadrant at 4wpi with uninfected and infected mice clustering separately, corresponding to the vector for phylum Patescibacteria. Mice from ODU1 and ODU2 generally clustered together, as did mice from uninfected and infected groups from all sites. For ODU sites a general shift in clustering in the same direction as vectors for phyla Bacteroidota, Cyanobacteriota, and Protobacteriae was noted.

### Effect of *S. mansoni* on the fecal microbiome composition at the family level

**Fig 3:**
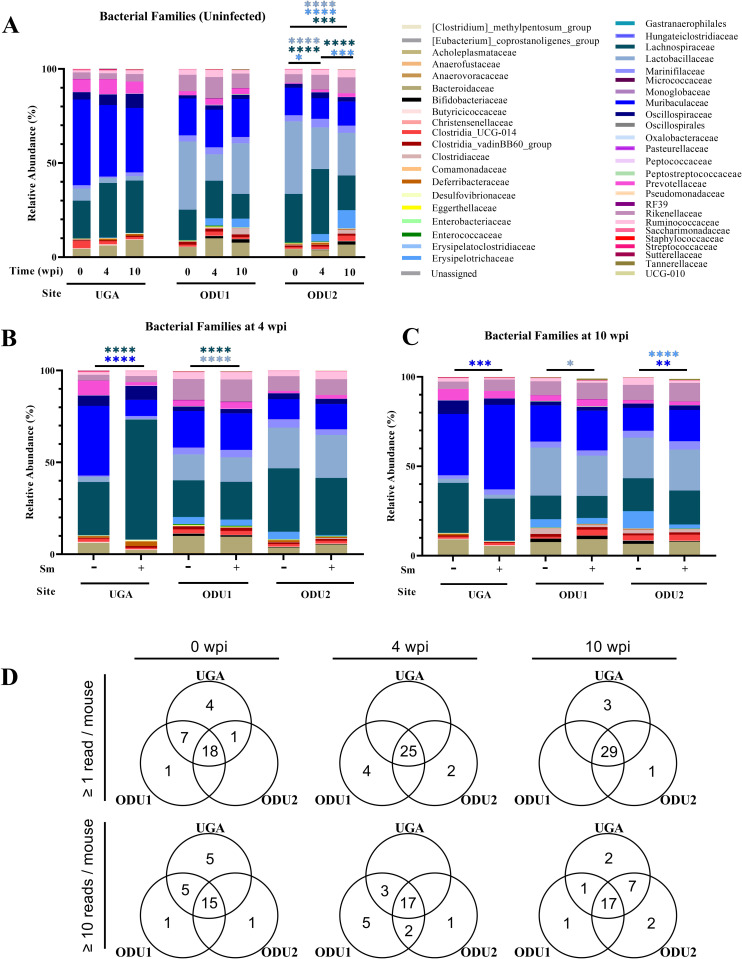
Relative abundances of bacterial families detected in fecal samples in bar plots. (A) The bar plots generated using GraphPad Prism, of relative abundance of the major bacterial families found in feces of uninfected mice at three different timepoints in three different experiments. The bar plots of relative abundance comparing infected and age-matched uninfected mice in three experiments for acute (B) and chronic (C) timepoints. (D) Venn diagrams showing the number of families that are shared among the majority (66%) of infected mice in the three different experiments at 0, 4 wpi and 10 wpi, respectively with and without setting a threshold of 10 reads. Findings were considered statistically significant at p < 0.05. *, **, and **** denote p < 0.05, p < 0.01, and p < 0.0001, respectively by one-way Anova using Sidak Test. The color of the significant asterisk indicates the respective family.

Forty-five total families were identified in all three experiments (Fig 3). Uninfected mice at UGA and ODU1 had non-significant age-related changes of their fecal microbiome composition at the family level at all timepoints (Fig 3A). ODU2 uninfected mice showed some age-related significant changes at 4 weeks: Erysipelotrichaceae and Lachnospiraceae showed significant increases while Lactobacilliaceae showed significant decreases. Also, ODU2 age-related significant changes were present at 10 weeks, Erysipelotrichaceae, Rikenellaceae, and Lactobacilliaceae increased significantly whereas Lachnospiraceae significantly decreased (Fig 3A).

Infected mice in the acute stage of infection (4 wpi) (Fig 3B) at UGA showed major significant differences in their compositions of Lachnospiraceae (+36.31%) and Muribaculaceae (-29.17%), compared to age-matched uninfected mice. At ODU1, infected mice had a significant increase in Lachnospiraceae and decrease in Lactobacillaceae (<10%). For ODU2, changes in infected mice compared to age-matched mice did occur but showed no statistical significance.

Infected mice in the chronic stage of infection (10 wpi) (Fig 3C) in all three experiments, showed significant changes in certain families’ abundance when compared to age-matched uninfected mice. Muribaculaceae increased significantly in UGA and ODU2 (<15%). Lactobacillaceae decreased in ODU1 infected mice. Finally, Erysipelotrichaceae decreased in ODU2 infected mice (<5%).

Out of the 45 identified families, 25 and 29 were present in the majority (66%) of infected mice’s fecal composition at acute and chronic stages, respectively. To minimize any findings to sequencing noise or artifacts, a threshold of ≥10 reads was established where 17 families were shared between the majority of infected mice’s fecal composition at both stages of infection. Comparing the infected stages to the pre-infection stage at 0 wpi, more families were shared between mice in the former (Fig 3D). During the acute infection, families identified in UGA infected mice were shared with both ODU1 and ODU2. ODU1 had 4 unique families prior to setting a threshold (Eggerthellaceae, Tannerellaceae, Streptococcaceae, Christensenellaceae) and 5 unique families with the threshold (Eggerthellaceae, Tannerellaceae, Gastranaerophilales, RF39, and Monoglobaceae). For ODU2 infected mice’s fecal samples, Bifidobacteriaceae was unique at ≥1 and Enterococcaceae was unique at both ≥1 and ≥10 to that experiment. During chronic infection, UGA had 3 pre-threshold unique families (Erysipelatoclostridiaceae, Clostridiaceae, and Enterobacteriaceae) and 2 post-threshold (Christensenellaceae and Clostridiaceae). ODU1 had RF39 as a unique family found in mice’s fecal samples after setting a threshold. Finally, chronically infected mice at ODU2 had Enterococcaceae unique to them pre-threshold and Streptococcaceae and Monoglobaceae post-threshold.

**Fig 4:**
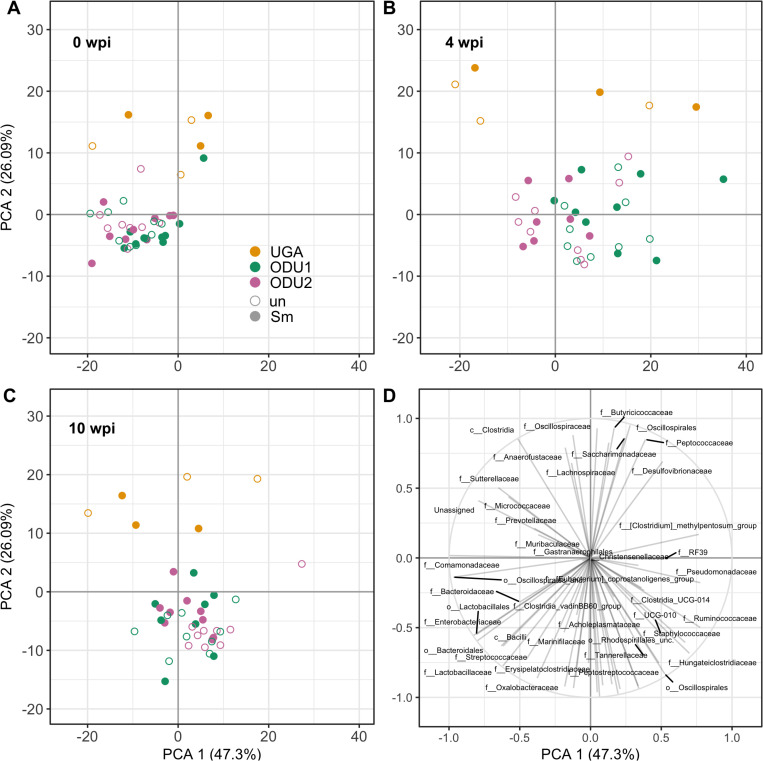
Relative abundances of bacterial families detected in fecal samples by Principal Component Analysis. Principal Component Analysis (PCA) based on the relative abundance of bacterial families found in the fecal microbiome. Infected (filled circle) and uninfected (open circle) mice at 0 (A), 4 (B), and 10 wpi (C) are shown from UGA (orange), ODU1 (bluish green), and ODU2 (reddish purple). (D) Major responsible families at all weeks.

Unlike the individual differences described above, when all mice were analyzed by Principal Component Analysis (PCA), there were no prominent differences associated with schistosome infection at the family level at ODU1 and 2 but it was observed in UGA mice, regardless of the time point (Fig 4).

### *Schistosoma mansoni*’s effect on alpha diversity of fecal microbiome in infected mice

**Fig 5:**
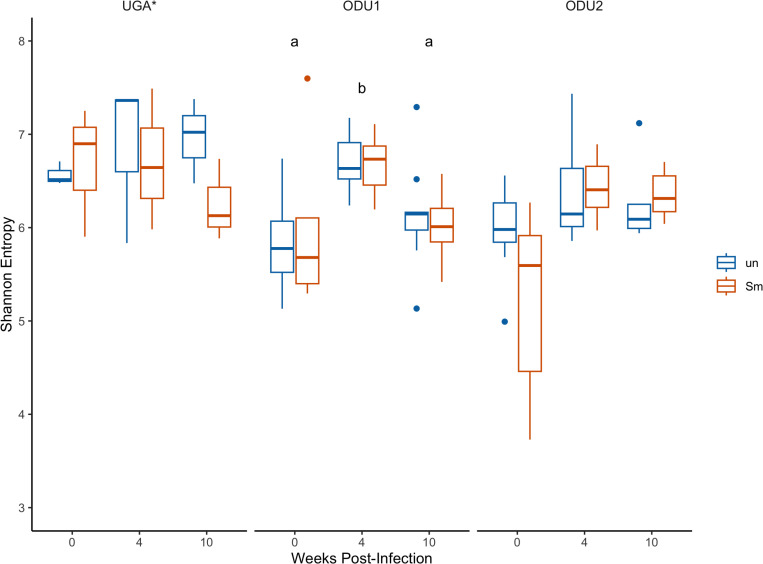
Alpha diversity of fecal microbiome. Boxplot showing the alpha diversity (Shannon Index) of fecal microbiome of uninfected (un-blue) and infected (Sm-red) mice at three different time points (0, 4, and 10 wpi) for three different experiments (UGA, ODU1, and ODU2). Data were analyzed with two-way Anova on rank-ordered data Tukey HSD post-hoc tests. Asterisk (*) by UGA denotes significantly higher alpha diversity than either ODU experiment. Differences among time points within ODU experiments are indicated by lowercase letters.

Alpha diversity in fecal microbiome among experimental groups was measured as Shannon entropy (Fig 5). Overall mice in the UGA experiments had higher alpha diversity than either ODU1 or ODU2 sites (Tukey post-hoc test; p = 0.004, p = 0.001, respectively), whereas alpha diversity did not differ by infection status (p = 0.67). Within sites, alpha diversity was significantly increased in the ODU1 experiment at week 4 compared to either week 0 or 10 (Tukey post-hoc test, p < 0.001), and was elevated at weeks 4 and 10 relative to week 0 in the ODU2 experiment.

### The effect of *Schistosoma mansoni* on beta diversity of fecal microbiome in infected mice

**Fig 6:**
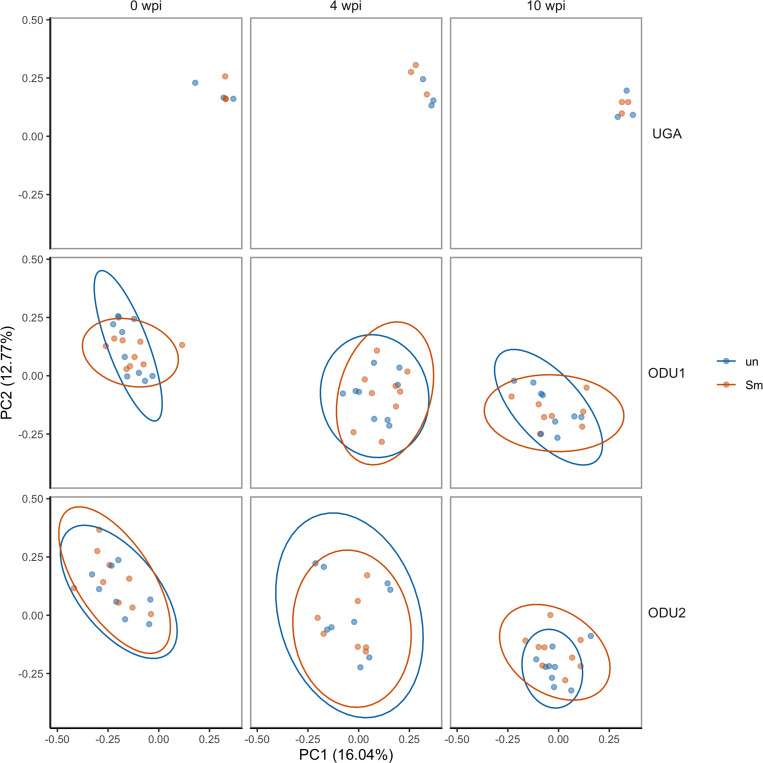
Beta diversity of fecal microbiomes by Principal Coordinate Analysis. Principal Coordinate Analysis (PCoA) of uninfected (un-blue circle) and infected (Sm-red circle) mice at three different time points (0 wpi, 4 wpi, and 10 wpi) for three different experiments (UGA, ODU1, and ODU2). The PCoA was plotted based on the beta diversity Bray-Curtis score of dissimilarity for each mouse such that every circle represents an individual mouse. Confidence ellipses are presented where sample size permitted with confidence level at 95% CI.

Unlike the individual family differences described in Fig 3, when all mice were analyzed for beta diversity with the Bray Curtis index, there were no prominent differences associated with schistosome infection in any of the three experiments, regardless of the time point (Fig 6). At all sites, uninfected and infected mice clustered together consistently over all time points. PERMANOVA analysis indicated that beta diversity was significantly affected by site. When observing the non-significant dispersion tests (S1 Table), there were significant effects on Bray-Curtis dissimilarity for three variables, infection status, weeks post infection, and the experiment.

## Discussion

Comparing the fecal microbiome of infected and uninfected mice between and within institutions proved to be a key component in identifying global changes that occur in fecal microbiome composition due to schistosomiasis. Initial fecal compositions varied by experiment; however, all the phyla and the majority of the families were shared between them. Age-related changes were minor in all 3 experiments. Mice at an acute stage of schistosome infection (4 wpi) typically display pro inflammatory T helper 1 (Th1) response occurring during the developmental stages of the parasite in the mammal host [19]. At this time point, we saw drastic changes in fecal composition at both the phyla and family levels at UGA, whereas this large change was not evident in the ODU1 and ODU2 mice. Potentially relevant was a murine parvovirus (MPV) infection that was detected in the experiments immediately following ODU1 and ODU2; if cryptic MPV infections were present and unidentified during ODU1 and ODU2, it could explain the lack of a major shift at the acute time point. The changes observed in the phyla (Bacillota and Pseudomonadota increasing) are concurrent with the literature where the ratio of Bacillota:Bacteriodota increases with pro-inflammatory conditions [20,21]. Pseudomonadota also increased consistently, regardless of the experiment, during the acute stage. This phylum has been corroborated with inflammatory conditions and acts as a biomarker for inflammation in the gut [22,23]. Regardless, we saw the majority of families (17–25/31) were shared by the majority of infected mice in all 3 experiments, 1 of which was not present or detectable in any of the uninfected mice. The patterns of change of the average abundance of phyla compositions in infected mice match the expectation and what is known about the plasticity of the fecal microbiome. Infected mice at a chronic stage of schistosome infection (10 wpi) typically display anti-inflammatory characteristics associated with an initial T helper 2 (Th2) response followed by a regulatory T response due to the parasites. This is an evasion technique of *Schistosoma spp* and other helminths to evade the host immune response [19]. At this time point, we saw differences in both phyla and family level compositions compared to uninfected mice; UGA was not statistically significant, likely attributed to the small sample size. Once again, the Bacillota:Bacteriodota ratio matched with the anti-inflammatory nature of this phase reflecting the presence of an anti-inflammatory response. This immune modulation contributes in addition to other mechanisms that *Schistosoma mansoni* employs to evade the immune system of the host. Notwithstanding, we saw the majority of families (17–29/33) were shared by the majority of infected mice in all 3 experiments. When analyzing overall composition and diversity, the largest visible differences were between institutions. However, there were some specific changes due to schistosome infection and these specific changes were reproducible, regardless of the institution at which the experiment was performed.

The families and phyla that changed throughout the experiments affected the homeostasis of fecal microbiome of mice at the different stages of schistosomiasis. The severity of the effect schistosomiasis on the host occurs mostly when eggs are sexually produced. This stage takes part in affecting the fecal microbiome composition as well as the systemic effect of the infection. Schistosomiasis was associated with the increase of bacterial gut composition in a pattern associated with its pathogenesis thereby affecting the infected mice compared to age-matched groups. This increases the parasitic burden in the host and provides risks for other co-morbidities. Focusing on the suspected families and potentially expanding these species-focused studies on human hosts, may allow progress in understanding the pathobiology of schistosome species as well as helminths sharing such characteristics and immune responses.

Our study is one of several addressing the specific changes and focusing on the minute details of the infected fecal microbiome [7,21,24]; however, the uniqueness of our study comes in comparing the environmental aspects and rigor into consideration. Comparing the findings of this study to other similar studies could provide more insights into repeatable patterns of fecal microbiome changes which is something our group is interested in performing. Even though the location of the experiment influenced the results observed, significant changes still occurred due to the infection at hand regardless of all the different variations between sites. This study reveals the importance of replicating the same microbiome study in separate locations to pinpoint the true contributing factors to microbiome changes. In addition, comparing the findings of similar groups could potentially provide an overview of any commonly observed effects that can be globally attributed to schistosomiasis and its pathogenicity.

## Supporting information

S1 Fig Alpha rarefaction curves of sequenced samples based on the Shannon entropy and sequencing depth. Individual curves represent individual samples.
(TIF)

S1 Table Summary of Adonis PERMANOVA of Bray-Curtis dissimilarity implemented in QIIME2. Findings were considered statistically significant at p < 0.05. Experiment refers to ODU1/ODU2 and UGA experiments.
(PDF)
