# Supplementary figures and images for: Schistosoma mansoni infection causes consistent changes to the fecal bacterial microbiota of mice across and within sites
Source: PLoS One. 2025 May 30;20(5):e0324638. doi: 10.1371/journal.pone.0324638 (PMC12124529; doi:10.1371/journal.pone.0324638)

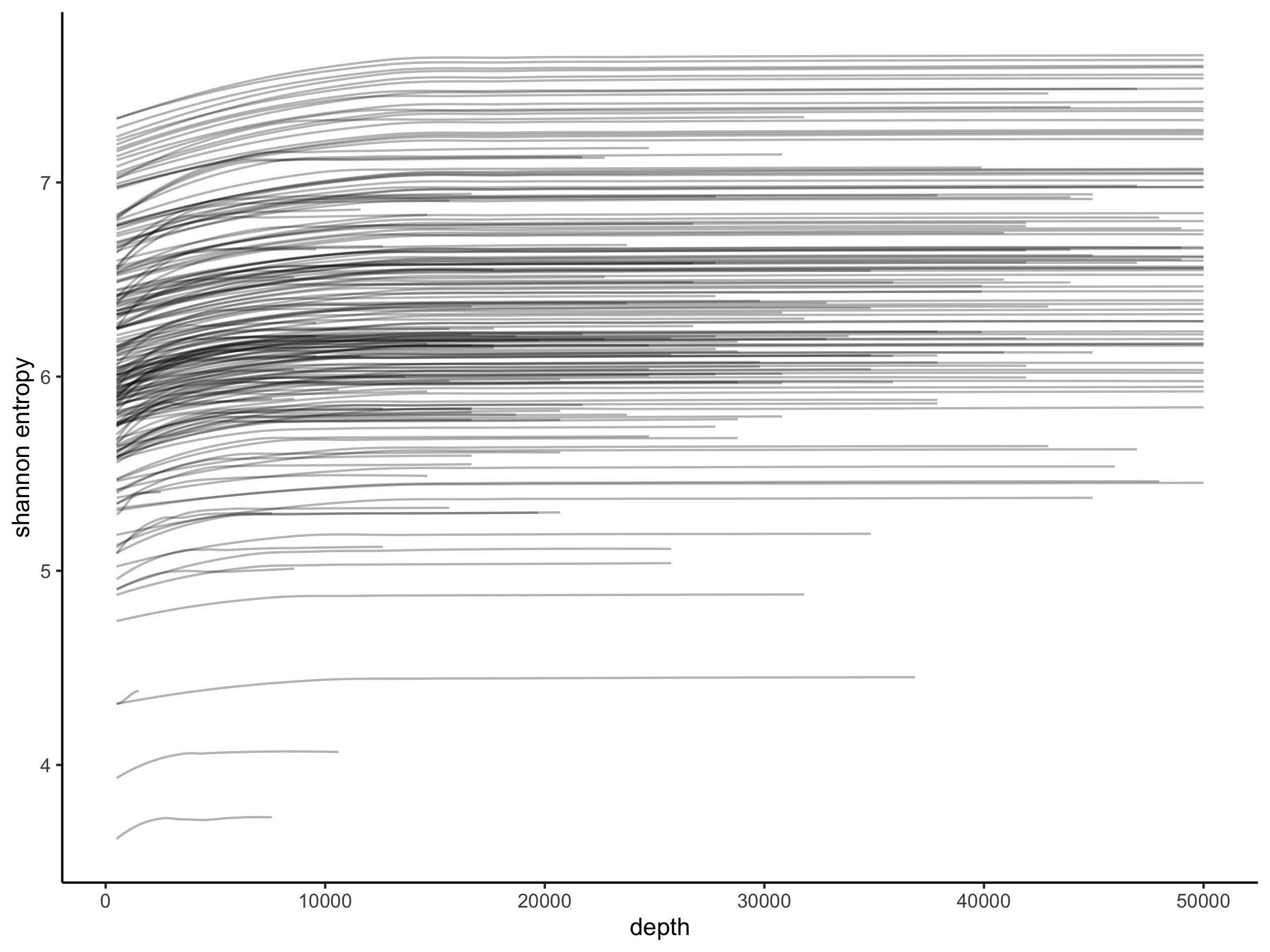

Supplement: S1 Fig — Individual curves represent individual samples. (TIF) [file pone.0324638.s001.tif]
