# Supplementary material for: Schistosoma mansoni infection causes consistent changes to the fecal bacterial microbiota of mice across and within sites
Source: PLoS One. 2025 May 30;20(5):e0324638. doi: 10.1371/journal.pone.0324638 (PMC12124529; doi:10.1371/journal.pone.0324638)
Supplement: S1 Table — Findings were considered statistically significant at p < 0.05. Experiment refers to ODU1/ODU2 and UGA experiments. (PDF) [file pone.0324638.s002.pdf]

S1 Table. Summary of Adonis PERMANOVA of Bray-Curtis dissimilarity implemented in QIIME2. Findings were considered statistically significant at $p < 0.05$. Experiment refers to ODU1/ODU2 and UGA experiments

	DF	F statistic	p-value
Experiment	2	12.71	0.001
Infection status	1	1.93	0.02
Collection time	1	11.11	0.001
Residuals	118	NA	NA
Total	122	NA	NA
